# Supplementary material for: Synergistic Antibiofilm Effects of Chestnut and Linden Honey with Lavender Essential Oil Against Multidrug-Resistant Otitis Media Pathogens
Source: Antibiotics (Basel). 2025 Feb 2;14(2):146. doi: 10.3390/antibiotics14020146 (PMC11851655; doi:10.3390/antibiotics14020146)
Supplement: Supplementary file 1 [file antibiotics-14-00146-s001.zip › antibiotics-3403173-supplementary.pdf]

Table S1. Antibiotic sensitivity of test bacteria (1-5) measured by disk diffusion assay. Data are presented as mean ± standard deviation (n=3).

	Inhibition zones (mm)				
**Antibiotics**	**1**	**2**	**3**	**4**	**5**
Amikacin	-	4.1±0.5	15.6±0.3	18.5±0.4	19.0±0.6
Ampicillin	7.5±0.2	8.5±0.8	16.2±0.3	6.5±0.5	4.0±0.3
Amoxicillin/ clavulanic acid	-	14.8±0.1	17.5±0.2	10.5±0.5	9.2±0.4
Ciprofloxacin	9.8±0.4	13.2±0.6	18.4±0.3	17.0±0.5	18.6±0.5
Ceftazidime	-	13.5±0.4	19.1±0.4	-	-
Erytromycin	-	-	3.2±0.3	-	17.5±0.2
Gentamicin	18.6±0.7	15.4±0.3	16.8±0.4	17.2±0.7	17.9±0.4
Imipenem	19.8±0.6	15.6±0.7	20.5±0.2	16.8±0.4	17.2±0.5
Levofloxacin	-	-	5.2±0.7	-	-
Oxacillin	3.8±0.2	-	-	-	-
Penicillin	6.7±0.5	-	-	-	-
Piperacillin/ tazobactam	-	12.5±0.5	17.4±0.9	-	-
Vankomycin	17.6±0.8	-	-	-	-

1: *Moraxella catarrhalis*; **2:** *Pseudomonas aeruginosa*; **3:** *Streptococcus pneumoniae*; **4:** *Haemophilus influenzae*; **5:** *H. parainfluenzae*

The diameter of the inhibition zones was measured in millimeter at 24 hours using a scale. An organism was interpreted as highly susceptible if the diameter of inhibition zone was more than 19 mm, intermediate if diameter was 15-18 mm and resistant if the diameter was less than 13 mm. The intermediate readings were considered as sensitive in the assessment of the data.
